# Recent Trends in the Chemical Modification of Polysaccharides for Food Packaging: A Review

**DOI:** 10.3390/polym18040529

**Published:** 2026-02-21

**Authors:** Paramabhorn Tosuwan, Hannah S. Leese, Christopher J. Chuck

**Affiliations:** 1 Department of Chemical Engineering, University of Bath, Claverton Down, Bath BA2 7AY, UK; 2 Centre for Bioengineering and Biomedical Technologies (CBio), University of Bath, Claverton Down, Bath BA2 7AY, UK

**Keywords:** food packaging, polysaccharide functionalization, functional packaging, barrier properties, biological activity, freshness monitoring

## Abstract

The environmental impact of petroplastics that do not readily biodegrade has intensified the search for sustainable packaging materials. Polysaccharides derived from plant and marine sources are biodegradable and renewable, but their hydrophilicity and weak mechanical and barrier properties limit their use in high-performance packaging. Chemical modification offers an effective solution by introducing hydrophobic or functional groups that enhance physicochemical performance, making modified polysaccharides strong candidates for sustainable packaging applications. This review provides a comprehensive overview of recent advances in the chemical modification and development of plant-based polysaccharides (starch, cellulose and its derivatives, and pectin) and marine-based polysaccharides (agar, carrageenan, alginate, and chitosan) for food packaging applications. Emphasis on how chemical modifications influence key functional properties relevant to sustainable packaging, including barrier performance, biological activities, and freshness-monitoring capabilities. Film fabrication techniques such as solution casting, extrusion, coating, and electrospraying are also discussed regarding their impact on material performance. Overall, the reviewed studies demonstrate that chemical modification can substantially enhance the functional properties of polysaccharides and enable active and intelligent packaging functionalities. While challenges related to food safety, scalable production, environmental impact, and real-world performance remain, chemically modified polysaccharides show strong potential as sustainable and functional materials for the next generation of food packaging.

## 1. Introduction

Food packaging plays a vital role in protecting food from environmental factors and contamination, helping to extend its shelf life and reduce food loss and waste. Conventional petroleum-based synthetic polymers such as polyethylene terephthalate, polypropylene, polystyrene, and polyvinyl alcohol have long dominated the packaging industry because of their durability, lightweight nature, water resistance, and low cost [1]. However, their nonbiodegradable nature has led to serious environmental concerns, including microplastic pollution and accumulation in landfills, which pose long-term risks to ecosystems and human health [2].

To address these issues, there is growing interest in developing sustainable alternatives that not only are able to biodegrade but also maintain essential packaging properties such as water resistance, low weight, and mechanical strength. Among various candidates, polysaccharides, derived from plant and marine sources, have emerged as promising materials for food packaging due to their abundance, low cost, low toxicity, biodegradability, biocompatibility, renewability, and reduced carbon footprint [3]. However, their inherent hydrophilicity and relatively poor mechanical properties limit their direct use in high-performance food packaging applications.

Chemical modification represents a promising strategy to overcome these limitations by introducing hydrophobic or functionally diverse groups into the polymer backbone. Such modifications enhance the physicochemical and functional properties of polysaccharides, improving their suitability for food packaging while maintaining environmental sustainability [4]. In recent years, substantial progress has been made in the development and application of these modified polysaccharide-based materials, reflecting their growing potential in sustainable packaging systems.

**Figure 1:**
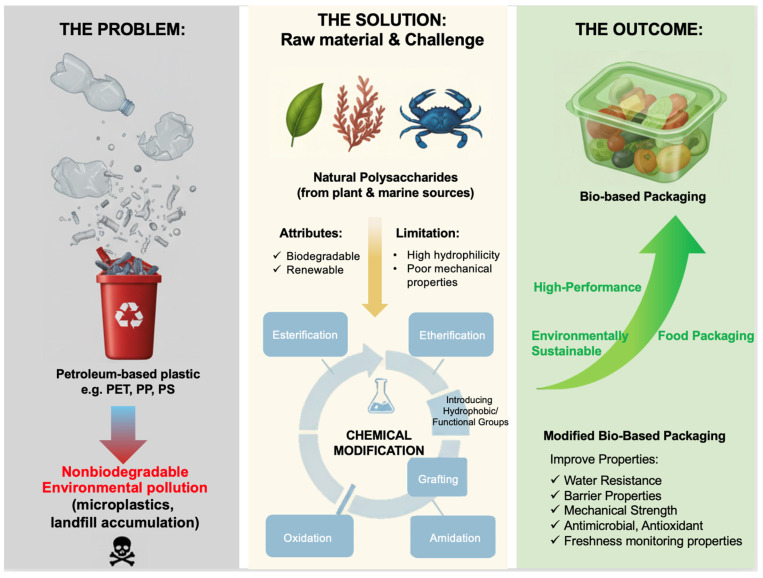
Overview of the motivation and strategies for developing sustainable food packaging materials from natural feedstocks.

This review provides a comprehensive overview of recent progress in the chemical modification of polysaccharides derived from plants and marine sources, with particular emphasis on reaction mechanisms, coupling strategies, and property optimization. The influence of these modifications on the barrier properties, biological activities, and freshness monitoring properties of polysaccharide-based packaging materials is critically discussed within the framework of sustainable material design (Figure 1). In addition, film and coating formation processes for improving material performance are examined. Finally, current challenges and future research directions for advancing polysaccharide-based systems toward intelligent and next-generation food packaging applications are evaluated.

## 2. Overview of Carbohydrates for Food Packaging

**Figure 2:**
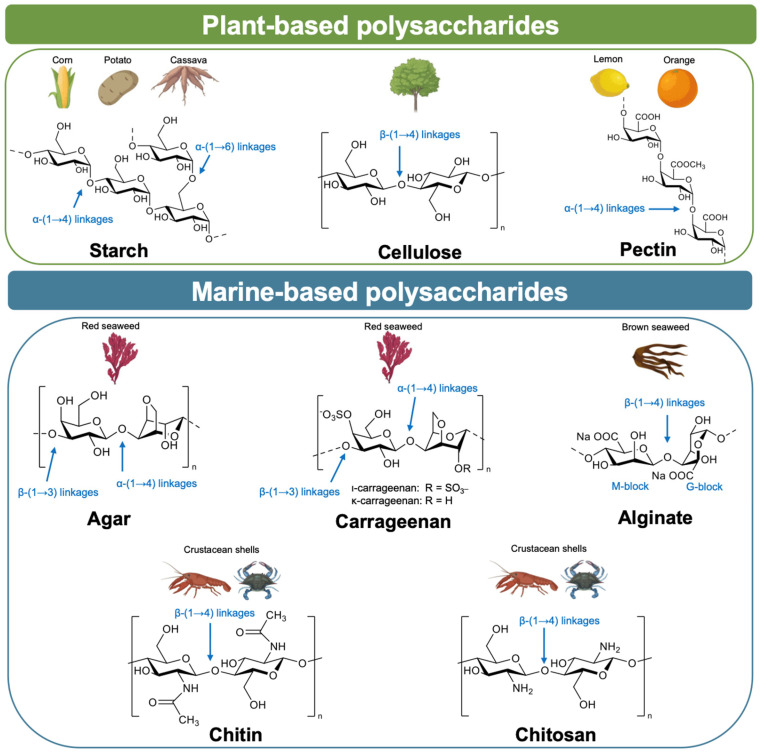
Chemical structures of major polysaccharides used in food packaging applications. Plant-based polysaccharides include starch (from crops), cellulose (from plants), and pectin (from fruit and vegetable cell walls). Marine-based polysaccharides include agar and carrageenan (from red seaweed), alginate (from brown seaweed), and chitin and chitosan (from crustacean shells).

Polysaccharides are among the most promising candidates for sustainable food packaging because they are abundant, renewable, and biodegradable. Their diverse chemical structures give rise to distinct physicochemical properties such as film-forming ability, transparency, gelling capacity, and barrier performance. However, the number of hydroxyl groups also makes them inherently hydrophilic, which often limits water resistance. Understanding the origin, structure, and intrinsic properties of different carbohydrate sources is therefore essential before exploring strategies for chemical modification. This section provides an overview of the most relevant polysaccharides derived from plants and marine sources for packaging applications, along with their structures (Figure 2). Emerging polysaccharide sources such as bacterial cellulose and fungal-derived chitin are also being explored for packaging applications [3]; however, this review focuses on the most widely studied plant- and marine-derived polysaccharides.

### 2.1. Plant-Derived Carbohydrates

Starch is a widely available biopolymer sourced from crops such as corn, potatoes, and cassava. It is composed of glucose units linked by glycosidic bonds, with two main structural components: amylose, a linear polymer of α-(1→4)-D-glucose, and amylopectin, a highly branched polymer containing both α-(1→4) and α-(1→6) linkages [5]. Natural starch typically occurs as granules of diverse forms and sizes, with diameters ranging from approximately 0.1 to 200 μm, depending on the botanical source [6]. Starch-based films are biodegradable and low-cost, but they are brittle and sensitive to moisture. Chemical modifications and grafting improve flexibility and water resistance, making starch a widely studied material for food packaging [7].

Cellulose is the most abundant natural polymer, sourced from plants, wood, or agricultural residues. It is a polysaccharide consisting of linear β-(1→4)-D-glucose chains forming crystalline microfibrils [8]. While native cellulose has limited solubility, its derivatives, such as carboxymethyl cellulose (CMC), microcrystalline cellulose (MCC), and nanocellulose, have excellent film-forming capacity and mechanical strength [9]. Its benefits include being renewable, cost-effective, non-toxic, biocompatible, biodegradable, and chemically stable, making it a valuable option for sustainable food packaging [10].

Pectin is an anionic polysaccharide extracted mainly from the peels of citrus fruits such as lemon, orange, lime, and apple pomace [11]. Pectin is a polysaccharide mainly composed of repeating (1→4)-linked α-D-galacturonic acid units. Its structure varies by plant source and typically includes three main domains: homogalacturonan (HG), rhamnogalacturonan I (RG-I), and rhamnogalacturonan II (RG-II) [12]. Pectin readily forms gels, which is why it is widely used in jams, fruit juices, desserts, dairy products, and jellies. More recently, it has been developed to expand its applications in active and biodegradable packaging [13].

### 2.2. Marine-Derived Carbohydrates

Agar is extracted from red seaweeds such as Gracilaria and Gelidium [14]. Agar is a mixture of two components: the linear polysaccharide agarose and a heterogeneous molecule called agaropectin. The chemical structure of agar consists of (1→4)-3,6-anhydro-α-L-galactose and (1→3)-β-D-galactose residues, with small amounts of esterified sulfate groups, generally not exceeding 6% (*w*/*w*) [15]. Agar readily forms transparent and flexible films, but its hydrophilicity leads to poor water resistance. Modifications are needed to enhance its hydrophobicity and packaging potential [16].

Carrageenan is a water-soluble polysaccharide extracted from red seaweed, consisting of alternating α-(1→4)-3,6-anhydro-D-galactose and β-(1→3)-D-galactose units with varying degrees of sulfation [15]. Its sulfate content influences solubility and functionality: λ-carrageenan (≈40% *w*/*w*) is highly sulfated and acts as a thickener without gelling ability, ι-carrageenan forms soft gels, and κ-carrageenan, with the lowest sulfate content (≈20% *w*/*w*), produces firm, brittle gels properties [17].

Alginate is a hydrophilic polysaccharide derived from brown seaweed, composed of (1→4)-linked β-D-mannuronic acid (M) and α-L-guluronic acid (G) residues. These residues are arranged in homopolymeric GG and MM blocks, as well as alternating MG blocks, which together form the polymer backbone [18]. In the presence of divalent cations such as Ca^2+^, alginate forms water-insoluble gels. Alginates with a low M/G ratio generate hard, brittle gels, and a high M/G ratio produces more elastic gels [19].

Chitin, the second most abundant natural polymer after cellulose, is biosynthesized by a wide range of organisms as a primary structural component of their shells and exoskeletons [20]. Chitin is found in crustacean shells, the primary large-scale source, as well as the exoskeletons of insects and the cell walls of various fungi [21]. Its molecular structure consists of N-acetylated D-glucosamine units linked through β-1,4 bonds, which can form a highly ordered crystalline arrangement, conferring enhanced mechanical strength and rigidity [22]. Owing to the presence of acetyl groups, chitin is hydrophobic and insoluble in most solvents; therefore, its applicability is greatly improved through deacetylation, which converts it into chitosan by replacing acetyl groups with hydroxyl (–OH) and amino (–NH_2_) groups under alkaline or enzymatic conditions [23]. Consequently, chitosan is composed of both deacetylated and acetylated D-glucosamine units connected via β-1,4 glycosidic bonds. The degree of acetylation significantly affects its chemical properties, such as tensile strength and solubility, as well as its biological characteristics, including bioavailability and biocompatibility [24].

## 3. Chemical Modification of Carbohydrates

**Figure 3:**
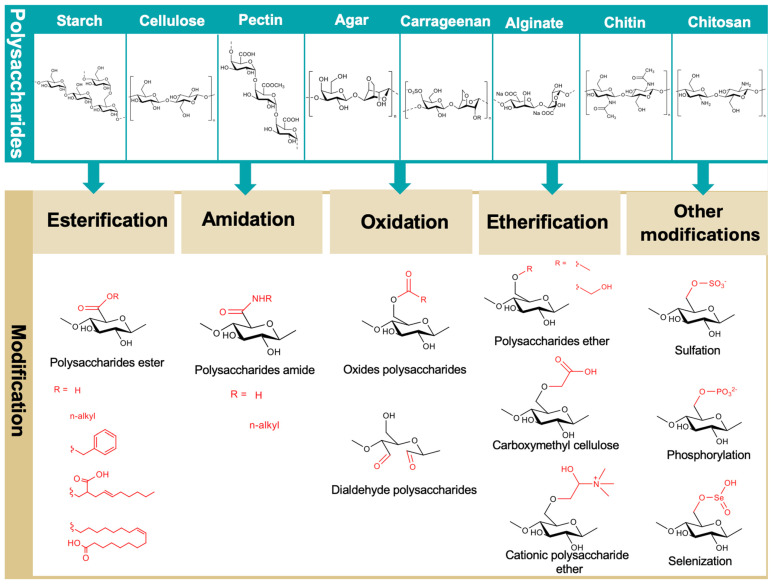
Common chemical modification strategies applied to polysaccharides to improve their functional performance in food packaging applications, including esterification, amidation, oxidation, etherification, and other modifications.

Polysaccharides contain abundant reactive groups, such as hydroxyl, carboxyl, and amine groups, which provide active sites for structural modification. In this section, we discuss key chemical modification strategies, including esterification, oxidation, amidation, etherification, and other modifications, which target these functional groups to tailor the polymer structure for specific applications and enhance the processability of polysaccharides (Figure 3).

### 3.1. Esterification

Esterification represents a crucial method for the chemical modification of polysaccharides, enabling precise control over their fundamental properties. This reaction involves the formation of a covalent ester linkage (-O-C(=O)-R) through the reaction of the polymer’s nucleophilic hydroxyl groups (-OH) with various acylating agents, typically carboxylic acids or their activated derivatives (anhydrides or acyl halides). The primary hydroxyl group at C6 is generally the most reactive, allowing the reaction to occur under mild conditions. For instance, benzoylated agar has been successfully synthesized under mild conditions using an aqueous slurry at 35 °C and 55 °C with a pH of 8.0–8.5, yielding derivatives with a degree of substitution (DS) ranging from 3% to 10% within one hour [25]. Additionally, reagents such as octenyl succinic anhydride are commonly used to create amphiphilic derivatives of polysaccharides via esterification, enhancing their interfacial activity, thermal resistance, and emulsifying stability [26]. To introduce a hydrophobic character, long-chain fatty acids can be employed to graft hydrophobic side chains. For example, oleic acid has been grafted onto cellulose surfaces through thermochemical esterification to improve the liquid water repellency of cellulosic paper [27]. A key consideration for this side-chain modification is that while it generally improves flexibility, hydrophobicity, and processability, excessively long chains can interfere with the polymer structure, ultimately reducing the thermal processability of the final material [28]. Overall, esterification provides a simple and efficient approach for functionalizing polysaccharides, conferring desirable properties such as water resistance, thermal stability, and interfacial activity. From a structure–property perspective, esterification outcomes are strongly dependent on DS and acyl chain length. Low-to-moderate substitution often increases hydrophobicity and water resistance, whereas excessive substitution or bulky/long chains can disrupt chain packing and reduce thermal processability or mechanical integrity [29].

### 3.2. Oxidation

Oxidation is one of the most widely applied chemical modification methods for polysaccharides, enabling the controlled introduction of reactive carbonyl and carboxyl groups into the polymer backbone. Polysaccharide chains contain two distinct hydroxyl groups, primary hydroxyls at C6 and secondary hydroxyls at C2 and C3. The primary hydroxyl group at the C6 position exhibits lower steric hindrance and can be selectively oxidized using various oxidizing systems, such as TEMPO/NaClO/NaBr (2,2,6,6-tetramethyl-1-piperidinyloxyl/sodium hypochlorite/sodium bromide) for cellulose, chitin, and carrageenan [30,31,32], H_2_O_2_ for agar [33], alkaline hexacyanoferrate(III) for carrageenan [34], and sodium hypochlorite for pectin [35]. The secondary hydroxyl groups at the C2 and C3 positions of polysaccharides can also be oxidized using sodium periodate, which cleaves the C2–C3 carbon bond to generate dialdehyde groups, as demonstrated for alginate [36] and pectin [37]. The oxidation-induced cleavage sites are highly reactive and serve as active centers for subsequent chemical modifications tailored to specific applications. For example, in alginate, the oxidized polymer can react with amines to form C=N (imine) bonds, which can then be reduced to introduce amino functionalities through reductive amination [38,39,40]. Oxidation provides reactive carbonyl/carboxyl groups that directly affect intermolecular interactions and enable secondary functionalization (e.g., Schiff-base formation or reductive amination). However, excessive oxidation—especially periodate-mediated backbone cleavage—may cause chain scission and reduced mechanical strength, making oxidation degree a critical design parameter [36].

### 3.3. Etherification

Etherification is an important chemical modification technique that introduces ether linkages (R–O–R′) into the polysaccharide backbone to tailor its properties. The reaction involves deprotonating hydroxyl groups with basic conditions, typically sodium hydroxide (NaOH), to generate reactive alkoxide intermediates (R–O^−^). These nucleophilic species then react with etherifying agents such as halides, epoxides, or alkenyl, forming covalent ether bonds via nucleophilic substitution or addition [41]. Compared to esterification, etherification requires more specific solvents than esterification because it typically involves the use of strong basic catalysts and polar aprotic media, such as dimethyl sulfoxide (DMSO), N,N-dimethylacetamide (DMAc) or aqueous alkaline systems (e.g., NaOH/urea), to effectively solubilize and activate the polysaccharide [42]. By selecting appropriate reagents, specific functional groups can be introduced, enabling precise control over properties such as solubility, viscosity, and surface activity, and thus tailoring the polysaccharide for targeted applications. For example, etherification of cellulose with methyl or hydroxy-ethyl groups improves the solubility of cellulose, which allows for water-based processing for applications in the food, biomedical, and cosmetics industries [43]. Moreover, etherification can modify the polysaccharide surface to introduce ionic functionalities. For instance, the carboxymethylation of polysaccharides such as cellulose and chitin involves the introduction of acidic groups onto the polymer backbone [44]. The resulting negatively charged polysaccharide ether has been studied as an alternative to conventional plastics in food packaging, helping to extend shelf life and preserve the quality of fruits and vegetables [45]. Etherification can also introduce positively charged quaternary ammonium groups into cellulose. These cationic cellulose ethers have been developed and used in highly conductive membranes [46] and as ion-exchange membranes in fuel cell applications [47]. Etherification often shows selectivity toward the more accessible C6 primary hydroxyl groups, while substitution at C2/C3 secondary hydroxyls is more sterically hindered and may require stronger activation conditions [48]. Etherification outcomes depend strongly on substituent type: alkyl ethers typically reduce polarity and improve water resistance, whereas functional ethers (e.g., carboxymethyl or quaternary ammonium groups) enhance hydrophilicity and ionic interactions.

### 3.4. Amidation

Amidation represents another effective chemical modification strategy for polysaccharides, allowing the introduction of amide functionalities that tailor their physicochemical properties. It is particularly effective for carboxyl-containing polysaccharides (e.g., alginate and pectin) because it selectively targets –COOH groups. This reaction involves the formation of a covalent amide linkage (–C(=O)–NH–) through the reaction of carboxyl or activated hydroxyl groups on the polysaccharide backbone with various amine-containing reagents. Amidation can proceed under mild conditions, particularly for carboxyl-containing polysaccharides such as alginate. Under acidic conditions (pH 3–5), alginate reacts with amines in the presence of coupling agents like EDC (1-Ethyl-3-(3-dimethylaminopropyl)carbodiimide) or DCC (N,N′-Dicyclohexylcarbodiimide) to yield amidated derivatives. Octylamine-functionalized alginate was prepared in aqueous medium (pH 3.4) using EDC at 35 °C for 24 h, achieving a DS of 10.8–30.3% [49]. Alternatively, 2-chloro-1-methylpyridinium iodide (CMPI) has been employed as a coupling agent for the amidation of alginate with amines of varying chain lengths to introduce hydrophobicity. CMPI has been found to provide higher DS compared to EDC (36 to 79%) by minimizing side reactions. However, when using CMPI, alginate must first be converted to its tetrabutylammonium (TBA) salt prior to the reaction [50]. Short-chain amines mainly affect polarity and intermolecular interactions, whereas long-chain alkyl amines improve water resistance by introducing nonpolar segments. However, excessive substitution can reduce solubility and hinder film formation [50]. Amidation thus offers a versatile approach to tailor polysaccharide properties, enabling the development of functional materials with enhanced performance for food packaging and other advanced applications.

### 3.5. Other Modifications

Grafting is a chemical modification technique that involves attaching polymer chains to the main polysaccharide backbone, either by coupling preformed polymer chains (grafting to) or by growing polymer chains directly from reactive sites on the backbone (grafting from). While chemical modifications such as esterification or etherification can improve polysaccharide properties, they are limited in achieving multi-functionality. In contrast, grafting attaches polymer side chains to polysaccharides, enabling the development of materials with enhanced and multiple *functions* [41]. Graft copolymerization is a key technique for chemically modifying chitosan to enhance antibacterial activity, as well as its chelating and complexation *abilities* [44]. *Various* initiator systems have been employed to facilitate this process, including potassium persulfate, ammonium persulfate, ceric ammonium nitrate, thiocarbonation–potassium bromate, potassium diperiodatocuprate, ferrous ammonium sulfate, enzymes, and γ-irradiation [51].

Sulfation is the process of adding sulfate groups to the polymer chain of polysaccharides by replacing hydroxyl groups under controlled conditions. The degree of sulfation depends on the molar ratio of reactants. Sulfated polysaccharides generally show higher antioxidant activity than their native forms [52]. Common methods for sulfation include chloro-sulfonic acid–pyridine [53], sulfuric acid [54], and sulfur trioxide–pyridine methods [55]. Similarly, phosphorylation is a chemical modification of polysaccharides in which phosphate groups replace hydroxyl groups in the polymer chain. This process enhances water solubility and alters the molecular weight and structure of the polysaccharides. For example, phosphorylated chitin was found to be water-soluble, representing a strategy to overcome this major drawback and enabling its broader use in biological and pharmaceutical applications where solubility is essential [56]. Phosphorylation can be performed under controlled conditions using phosphate [57], phosphoric acid [58], or phosphorus oxychloride [59] as effective and relatively safe phosphorylating agents.

A secondary reaction is also possible. For example, the Schiff-base reaction involves the reaction of an amine group (–NH_2_) with an aldehyde or ketone (C=O) to form an imine bond (C=N). In polysaccharides such as chitosan or modified aminated carbohydrates, this reaction allows the amino groups to interact with carbonyl compounds, forming crosslinked structures that enhance stability and make them suitable for bioplastic applications [60,61].

Further to organic reactions, inorganic components have also been incorporated into a modified polymer. For example, selenization is a chemical modification of polysaccharides in which selenium atoms are incorporated into the polymer chain. It can be achieved using various methods, including the glacial acetic acid–sodium selenite [62], nitric acid sodium-selenite method [63], and selenium oxychloride methods [62].

Several other methods, such as thiolation, imidization, and sulfonylation, have also been employed for the modification of polysaccharides [52,64]. Although these techniques are not widely used, they have made valuable contributions to enhancing the physicochemical and pharmacological properties of value-added polysaccharides. Alternative modifications are largely governed by the balance between introducing new functional groups and preserving the native polysaccharide backbone. For example, grafting can impart multifunctionality, while sulfation and phosphorylation selectively substitute hydroxyl groups to increase polarity and solubility but may also raise water uptake, potentially compromising moisture barrier performance at high substitution levels. Schiff-base reactions enable convenient crosslinking between aldehyde-bearing polysaccharides and amine-containing polymers to form denser networks.

## 4. Functional Properties Relevant to Food Packaging

For polysaccharide-based materials to move beyond the laboratory and into real food packaging applications, their functional performance must meet the demands of modern packaging systems. Chemical modifications not only alter the polymer backbone but also unlock new properties that can rival or complement conventional plastics [65]. Key attributes, such as mechanical strength, barrier properties against water, oxygen, and fats, hydrophobicity, thermal stability, and biodegradability, directly influence how well these materials protect and preserve food. At the same time, antioxidant and antimicrobial activities open possibilities for active packaging, while morphological characteristics and food-grade safety ensure practicality and regulatory compliance. In this section, we explore how modifications of polysaccharides influence their functional properties with a focus on their relevance to sustainable food packaging.

### 4.1. Functional Properties Achieved Through Esterification

Esterification is one of the most widely studied chemical modification techniques for enhancing the performance of polysaccharide-based materials. Through the introduction of ester groups, properties such as hydrophobicity, mechanical strength, and barrier performance can be tailored to meet specific packaging requirements. Table 1 summarizes representative studies on esterified polysaccharides, highlighting the ester compounds used, the modification method, the functional improvements achieved, as well as the associated advantages and existing challenges. Common trends include using long-chain acids or anhydrides (e.g., lauric acid, maleic anhydride, and octenyl succinic anhydride) to improve hydrophobicity, water, and gas barrier properties. Mild and direct esterification methods, or the use of coupling agents like DCC/DMAP, are often employed to preserve polymer integrity. Bioactive compounds like anthocyanins, keratin, or essential oils can add antioxidant, antimicrobial, or pH-sensitive functions. Advantages include biodegradability, eco-friendly reagents, and simple processing, while challenges involve reduced mechanical flexibility, thermal sensitivity, non-uniform degradation, and occasional sensory issues, reflecting trade-offs for practical food packaging.

In general, the type of esterifying agent strongly influences the resulting structure–property relationships. Esterification using longer alkyl chains (e.g., lauric acid or octenyl succinic anhydride) tends to provide greater improvements in hydrophobicity and water resistance because the introduced nonpolar segments reduce surface polarity and limit water–polymer interactions. In contrast, multifunctional short-chain acids (e.g., citric acid and oxalic acid) often contribute to barrier enhancement primarily through crosslinking effects that restrict chain mobility, rather than through strong hydrophobicity. In addition, esterification that simultaneously introduces bioactive or indicator compounds (e.g., anthocyanins, essential oils, or keratin) enables multifunctional films with antimicrobial, antioxidant, or freshness-monitoring properties, although some studies report increased water vapor permeability or reduced sensory acceptability [66,67,68]. Overall, the most promising approaches appear to be mild esterification routes (direct esterification or coupling-assisted reactions) combined with controlled substitution and functional additives, as they offer a practical balance between improved barrier performance, mechanical stability, and added intelligent/active functions.

**Table 1:** Representative studies on esterified polysaccharides: ester compounds, modification method, functional improvements, advantages, and challenges.

Polysaccharides	Secondary Compound	Modification Method	Functional Properties	Advantages	Challenges	Reference
Hemicellulose	Lauric acid	Esterification via DCC and DMAP coupling agents	Improved hydrophobicity	Transparency, recyclable, biodegradable	Sensitivity to high temperatures (>70 °C)	[4]
κ-Carrageenan	Maleic anhydride	Direct esterification	Improved UV and water barrier properties	Energy and processing savings due to low dissolving, gelling, and melting temperatures	Reduction in tensile strength and thermal stability	[69]
Cellulose	BTDA */Cellulose nanocrystals (CNSs)	Esterification with BTDA; CNCs incorporated to reinforce matrix	Improved tensile strength	Bio-based materials	Reduction in elongation at break	[70]
Cellulose	Citric acid	Direct esterification	Improved moisture barrier properties	Environmentally friendly and non-toxic substrates	Rigid structure-restricted flexibility	[71]
Nanocellulose	Oxalic acid	Acid-catalyzed esterification	Improved water barrier properties	Extended shelf life for fruits	Degradation risk with high oxalic acid ratio	[72]
Nanocellulose	Butyric acid, hexanoic acid, octanoic acid	Esterification via DMAP coupling agent	Oxygen and water vapor barrier properties	Biodegradability, scalable processing	Decreased thermal stability	[73]
Cellulose	Citric acid, keratin	Esterification using citric acid as linker	Antimicrobial and antioxidant activities	Biodegradation	Non-uniform degradation patterns	[74]
Cellulose nanofibers	Vanillic acid and cinnamic acid	Esterification via thionyl chloride and EDC	Antioxidant and antimicrobial activities	Sustainable and safe base material	May contain metal contamination	[75]
Starch	Citric acid, polyvinyl alcohol, carboxycellulose	Esterification using citric acid as linker	Improved water-resistant, gas barrier and antibacterial activities	Litchi and plum preservation, biodegradation	-	[76]
Starch	Maleic acid, polyvinyl alcohol	Esterification using citric acid as linker	Water and gas barrier properties	Simple and safe preparation process	Crosslinking is ineffective at low maleic acid concentrations.	[77]
Starch	Octenyl succinic acid anhydride, honey-bee products	Direct esterification	Improved hydrophobicity and antimicrobial activity	Higher thermal stability	Reduced sensory acceptability	[66]
Starch	Purple corncob anthocyanin, tangerine peel essential oil	Ultrasonic esterification of starch with dodecenyl succinic anhydride to stabilize essential-oil pickering emulsions.	Antimicrobial indicator	Monitoring pork freshness	Increased water vapor permeability	[67]
Strach	Red cabbage anthocyanin	Dual chemical modification (acetylation–phosphation, oxidation–acetylation, oxidation–hydroxypropylation).	water-resistance, water vapor barrier, pH-sensitive properties	Color reversibility	Reduced elongation	[68]
Alginate	Octenyl succinic anhydride	Direct esterification	Improve water and water vapor barrier	Higher tensile strength	Reduced extensibility	[78]
Agar	Benzoic acid derivatives	Esterification via EDC and DMAP coupling agents	Antioxidant and bacteriostatic properties	Improve biological properties, fish preservation	Further research is needed to improve the long-term performance of the coating	[79]

* BTDA = 3,3′,4,4′-Benzophenone tetracarboxylic dianhydride. DCC = N, N′-dicyclohexylcarbodiimide. DMAP = 4-dimethyl-aminopyridine. EDC = 1-ethyl-3-(3-dimethylaminopropyl) carbodiimide.

### 4.2. Functional Properties Achieved Through Oxidation

Oxidation is a versatile chemical modification technique that introduces aldehyde and carboxyl groups onto the polysaccharide backbone, creating reactive sites for further functionalization [80]. This reaction improves crosslinking ability, enhances mechanical strength, improves both antioxidant and antimicrobial activities and reduces water absorption, thereby contributing to better barrier and structural properties in food packaging. Table 2 summarizes recent studies on oxidized polysaccharides, highlighting the compounds bound to the polysaccharide chains, the modification method, their functional improvements, advantages and limitations. Notable trends include widespread use of TEMPO-mediated oxidation for cellulose and nanocellulose, often combined with bioactive agents (e.g., silver nanoparticles, curcumin, and essential oils) to impart antimicrobial, antioxidant, or UV-blocking properties [81,82]. Periodate oxidation is mainly applied to improve water and oxygen barrier performance, sometimes with biopolymers like gelatin. While these methods offer eco-friendly, biodegradable materials with improved functionality, challenges such as acid sensitivity, metal toxicity, and barrier trade-offs remain, requiring further optimization for food applications.

From a structure–function perspective, oxidation is often used not only to improve barrier performance directly, but also to create a chemically reactive “gateway” polysaccharide for subsequent functionalization. Carboxyl-rich oxidized cellulose/nanocellulose can serve as an effective reinforcing and reactive phase, enabling covalent coupling of antioxidants and antimicrobials through carbodiimide chemistry (EDC/NHS) and improving compatibility within composite matrices [83,84]. In contrast, aldehyde-bearing oxidized polysaccharides promote network formation through Schiff-base reactions, which can generate dense structures and improved water/oxygen barrier performance [85]. Table 2 further highlights that the choice of incorporated antimicrobial agent is a key practical issue: although metal nanoparticles provide strong antimicrobial effects, potential migration and toxicity concerns are driving increasing interest in non-metal alternatives (e.g., phenolics, essential oils, or encapsulated bioactives). Overall, oxidation strategies appear most promising when combined with stable crosslinking and safer bioactive systems to achieve durable functionality without compromising barrier performance or safety.

**Table 2:** Representative studies on oxidized polysaccharides: compounds incorporated, modification method, functional improvements, advantages, and challenges.

Polysaccharides	Secondary Compound	Modification Method	Functional Properties	Advantages	Challenges	Reference
Cellulose	Kafirin protein	TEMPO-oxidized cellulose used as a crosslinker in kafirin matrix	Improved water barrier properties	Eco-friendly preparation process	Alkaline instability	[86]
Cellulose	-	Oxidation by sodium periodate	Improved oxygen barrier	Green synthesis and processing	Limited solubility	[87]
Nanocellulose	Silver nanoparticles, grape seed extracts	TEMPO-oxidized nanocellulose and blend with silver and grape seed extract	Antioxidant and antimicrobial activity	Bio-based materials	Sensitive to acidic conditions, risk of Ag+ toxicity	[81]
Cellulose	Silver nanoparticles	TEMPO-oxidized cellulose with added silver nanoparticles	Antimicrobial activity	Strong stabilization and simple synthesis	Risk of Ag+ toxicity	[82]
Nanocellulose	Zeolitic Imidazolate Framework-8 (ZIF-8), carvacrol	TEMPO-oxidized nanocellulose, modified with ZIF-8 to encapsulate carvacrol	Antimicrobial activity	Fruit preservation	Sensitive to acidic conditions	[88]
Cellulose	Gelatin, gallic acid	Periodate-oxidized cellulose, Schiff-base–gelatin, gallic acid added	Water resistance and barrier properties	Biodegradability and mold resistance	Sensitive to acidic conditions	[85]
Chitosan	Phenol red	Hydrogen peroxide-oxidative hydrolysis of chitosan and Mannich reaction with phenol red	pH-responsive	Monitored shrimp freshness	Decreased thermal stability	[89]
Starch	Sorbitol and glycerol	Sodium hypochlorite oxidized starch with added sorbitol and glycerol	Improves the physicochemical characteristics	Bio-based materials	Further studies needed for real food systems.	[90]
Nanocellulose	Oregano essential oil (OEO), polyethyleneimine (PEI)	TEMPO-oxidized cellulose nanocrystals, grafted with PEI via EDC/NHS, with added OEO	Antimicrobial and antioxidant properties	Barrier property of water vapor	Reduced the barrier property of oxygen	[83]
Chitosan, cellulose nanofiber	Curcumin	TEMPO-oxidized cellulose nanofiber grafted with curcumin via EDC/NHS esterification, then incorporated into chitosan films by solution casting	Antimicrobial and UV-blocking properties	Inhibit oxidation and extend the shelf-life	Cellulose increased oxygen transmission	[84]

EDC = 1-ethyl-3-(3-dimethylaminopropyl) carbodiimide. NHS = N-Hydroxysuccinimide.

### 4.3. Functional Properties Achieved Through Etherification

Etherification is employed to modify polysaccharides by substituting hydroxyl groups with alkyl or carboxymethyl groups, thereby altering surface polarity and solubility. This process improves hydrophobicity, flexibility, and film transparency, critical attributes for sustainable packaging materials. Table 3 summarizes representative studies on etherified polysaccharides, highlighting the compounds incorporated, the modification method, their functional enhancements, advantages, and challenges. Notable trends in the etherification of polysaccharides, often via alkali-activated reactions with epoxides or alkyl halides, include the enhancement of water resistance, mechanical strength, thermal stability, and functional properties such as antimicrobial, antioxidant, or pH-responsive activity. Common advantages include sustainability, non-toxicity, and compatibility with food-grade applications, while challenges involve processability, dispersion, homogeneity, and sometimes reduced mechanical performance or limited long-term stability. Importantly, etherification enables two distinct design directions for packaging films. Hydrophobizing etherification (alkyl substitution) is more suitable when the primary goal is moisture resistance [91], whereas functional etherification (e.g., carboxymethylation or cationization) is more advantageous for improving interfacial compatibility and enabling the incorporation of active agents. This is reflected in Table 3, where carboxymethylated or cationized derivatives are frequently combined with polyphenols, ZnO, or anthocyanins to achieve antimicrobial, antioxidant, UV-blocking, or freshness-indicating functions [92,93,94,95,96]. However, these multifunctional systems often require careful optimization of substitution degree and additive loading to avoid sacrificing film integrity. Overall, etherification strategies that combine moderate substitution with controlled additive incorporation appear most promising for balancing processability, mechanical performance, and active/intelligent packaging functionality.

### 4.4. Functional Properties Achieved Through Alternative Modifications

Beyond the major modification routes, polysaccharides can also be functionalized through additional covalent chemical transformations (such as amidation and quaternization) or non-covalent strategies (such as blending). These methods can significantly influence physicochemical and biological properties, including antioxidant, antimicrobial, and water-resistance performance. Table 4 compiles examples of alternative modification strategies, highlighting the compounds incorporated into polysaccharide-based systems, their potential advantages, and current challenges for food packaging applications. Trends include the use of amidation, quaternization, and grafting (covalent modifications), as well as blending (non-covalent modifications), to improve hydrophobicity, water vapor barrier, mechanical strength, and bioactive properties such as antimicrobial and antioxidant activity. Incorporation of secondary compounds, ranging from alkyl amines, vanillic acid, and lignin to metal–organic frameworks, enables multifunctional features including pH responsiveness, light/UV blocking, and freshness monitoring. Advantages include biodegradability and suitability for food preservation, whereas challenges include processability limitations, potential toxicity of unreacted reagents, thermal degradation, and higher production costs. Overall, these alternative strategies differ mainly in whether they involve permanent covalent modification of the polysaccharide backbone or rely on non-covalent interactions. Covalent approaches such as amidation, quaternization, and grafting introduce new functional groups and can provide more durable improvements in antimicrobial activity, barrier performance, and water resistance; however, they may require stricter control of reaction conditions and purification to minimize residual reagents and ensure food-contact safety. In contrast, blending strategies provide a simpler and more scalable route to improve film formation, mechanical properties, and surface uniformity through hydrogen bonding or polyelectrolyte complex formation (e.g., chitosan/κ-carrageenan systems).

### 4.5. Barrier Properties

Barrier properties are crucial in food packaging, as moisture, gases, and light can accelerate spoilage and reduce shelf life. Polysaccharides, with their tunable chemical structures, provide a versatile platform for developing materials with improved resistance to water, oxygen, and light [111]. Through targeted chemical modifications, their barrier performance can be significantly enhanced. Depending on the protection needed, barrier properties are typically categorized into water, gas, and light barriers.

#### 4.5.1. Water Barrier

Polysaccharides naturally absorb moisture due to their abundant hydroxyl groups, which limits their effectiveness in humid environments. Therefore, improving water barrier performance is crucial for expanding their use in food packaging. Chemical modification is an effective strategy to reduce hydrophilicity and improve resistance to water penetration. Introducing hydrophobic substituents has been particularly successful in enhancing the water barrier performance of polysaccharide-based films. For instance, long-chain fatty acid esterification reduces the availability of hydrophilic sites and introduces hydrophobic groups, thereby decreasing water uptake and water vapor transmission [4]. This improvement is often attributed to two factors: (i) the substitution of hydroxyl groups reduces hydrogen bonding with water, and (ii) the introduction of nonpolar chains increases surface hydrophobicity and disrupts water diffusion pathways [112]. Oxidation can also enhance water barrier properties when it leads to crosslinking. TEMPO-mediated oxidation and periodate oxidation have been shown to increase cellulose reactivity, crosslinking with gelatin to improve mechanical properties and water resistance [85]. Similarly, amidation and etherification may improve water resistance by introducing bulky or hydrophobic moieties that create steric hindrance and reduce the polymer’s affinity for water [91]. For example, grafting aliphatic amine hydrophobic chains (C12–C18) to alginate increased the hydrophobicity of alginate to form a water-resistant coating layer for food packaging [50].

#### 4.5.2. Water Vapor Barrier

A stringent water vapor barrier is particularly challenging to achieve because most hydrophilic polysaccharides, such as chitosan, alginate, and starch, readily absorb moisture. Insufficient water vapor resistance can compromise food quality by accelerating microbial growth, inducing texture changes, and shortening shelf life. Based on water vapor permeability, films are classified from poor to very high barrier performance as follows: poor (>3000 g·μm/m^2^·day·kPa), low (100–300 g·μm/m^2^·day·kPa), medium (400–1000 g·μm/m^2^·day·kPa), high (40–400 g·μm/m^2^·day·kPa), and very high (<40 g·μm/m^2^·day·kPa) [113]. Moisture transmission rate requirements vary depending on the type of food being protected. Strategies to enhance water vapor barrier properties include chemical modification and the incorporation of hydrophobic moieties. For instance, the introduction of vanillic acid to chitosan via amidation and esterification has been shown to improve water vapor barrier performance [102]. In low-molecular-weight chitosan films, the water vapor transmission rate decreased from 51.46 ± 1.98 g/m^2^·h to 46.70 ± 0.90 g/m^2^·h after vanillic acid modification. This reduction is attributed to the formation of covalent ester and amide bonds, which decrease the number of hydrophilic groups, increase the hydrophobicity of the film, and create a denser polymer network that limits water vapor diffusion. The vanillic chitosan material can be used to preserve cherry tomatoes at 25 °C for 10 days. In addition to chemical modification, blending polysaccharides with hydrophobic components, such as Zein, lipids, or waxes, can further improve water vapor resistance by creating tortuous pathways for moisture diffusion [114]. These approaches highlight the complexity and importance of optimizing water vapor barrier properties for the preservation of food quality and shelf life.

#### 4.5.3. Gas Barrier

Gas barrier performance is critical for maintaining food quality, as oxygen strongly influences oxidation reactions, microbial growth, texture changes, and overall shelf life. Based on oxygen permeability (cm^3^·μm/m^2^·day·atm), films are classified from poor to very high barrier performance as follows: poor (>40,000), low (4000–40,000), medium (400–4000), high (40–400), and very high (<40) [113]. Polysaccharides such as nanocellulose exhibit excellent gas barrier properties (oxygen permeability = 118 cm^3^·μm/m^2^·day·atm) due to their dense hydrogen-bonded networks and high crystallinity (72–80%), which restrict the mobility of polymer chains and significantly limit gas diffusion [115,116]. Despite these advantages, the gas barrier performance of many natural polysaccharides can be further enhanced through chemical modification or structural tailoring. Such modifications often reduce free volume, increase matrix compactness, or introduce hydrophobic moieties that impede oxygen permeation. For example, introducing short-chain fatty acids into cellulose nanofiber films via esterification has been shown to reduce oxygen transmission rates [73]. These strategies highlight the potential of tailored polysaccharide-based materials to achieve improved gas barrier properties for food packaging applications.

#### 4.5.4. Light Barrier

Light barrier properties are essential for protecting photosensitive foods, such as milk and edible oils, which can undergo nutrient loss, lipid oxidation, discoloration, and off-flavor formation when exposed to light, ultimately reducing shelf life and sensory quality [117]. Most native polysaccharides have limited light-blocking capacity because they lack strong UV-absorbing groups, making the addition of functional additives necessary to improve their protective performance [118]. Chemical modification provides a practical approach to enhancing light shielding by incorporating UV-absorbing or light-scattering functionalities. For instance, grafting inorganic nanoparticles like ZnO [95] and organic compounds like lignin and cellulose onto polysaccharide chains can introduce strong UV absorption [84,119]. Similarly, esterification with maleic anhydride has been reported to significantly reduce UV transmittance in κ-carrageenan-based films [69]. The native κ-carrageenan films showed a moderate reduction in transmittance (dropping to 62% in the 236–300 nm range), whereas the modified films exhibited nearly complete UV absorption (transmittance dropping to 0%). This effect is attributed to the introduction of maleic anhydride groups, which form conjugated double bonds that enhance UV absorption. These modified materials not only protect light-sensitive foods but can also help preserve color and nutritional quality, making them suitable for packaging oils, fruit products, and nutraceutical formulations.

### 4.6. Biological Activity (Antioxidant and Antibacterial Properties)

Biological activities such as antioxidant and antibacterial functions play a crucial role in active food packaging, helping delay oxidation, suppress spoilage, and extend shelf life. While most native polysaccharides possess limited inherent activity, chemical modification enables the incorporation of functional groups that significantly enhance their bioactive performance [120]. Antimicrobial activity targets spoilage microorganisms and foodborne pathogens such as *Listeria monocytogenes*, *Salmonella*, and *Escherichia coli*, which contribute significantly to global food waste [121]. Packaging materials achieve this activity by incorporating inorganic agents (e.g., metal oxide and silver nanoparticles), organic antimicrobials, or natural compounds that disrupt microbial metabolism. Inorganic antimicrobial agents provide long-lasting activity by slowly releasing metal ions that disrupt bacterial membranes and damage essential cellular components [122]. For example, ZnO–chitosan films that effectively inhibit bacterial growth extended mango shelf life to 11 days [96]. Organic antimicrobials, such as chitosan and phenolic-rich essential oils, can inhibit bacterial protein or cell-wall synthesis, disrupt energy production, and interfere with cellular metabolic processes [123,124]. Antioxidant activity protects food components prone to oxidation, such as lipids, proteins, and polyphenols, thereby delaying browning, rancidity, and sensory deterioration. While synthetic antioxidants like butylated hydroxyanisole (BHA) and butylated hydroxytoluene (BHT) face safety concerns, natural antioxidants from plant extracts and essential oils are preferred for their biocompatibility [116]. For instance, polyphenols extracted from walnut green husk incorporated into curdlan and cellulose films have produced packaging with strong antioxidant performance (up to 79% DPPH scavenging) [93].

### 4.7. Freshness Monitoring Properties

While antimicrobial and antioxidant systems help inhibit spoilage, the next generation of packaging adds the ability to detect and signal changes in food freshness. Materials with monitoring properties respond to variations in pH, temperature, humidity, gas composition, or microbial activity, complementing biologically active packaging by providing diagnostic information on early signs of deterioration. Chemical modification of polysaccharide-based freshness-monitoring materials typically produces visual signals, most commonly color changes, that reflect pH variations generated during microbial metabolism, enabling effective real-time monitoring of food quality during storage. Natural pigments such as anthocyanins are frequently used to create pH-responsive labels [110]. For example, anthocyanins extracted from purple corncob were incorporated into a starch-based film to monitor pork freshness, showing a distinct color shift from red to yellow as pH increased, thereby distinguishing fresh from spoiled meat [67]. Similarly, anthocyanins from black wolfberry and red cabbage have been incorporated into polysaccharide matrices to track freshness in chicken and shrimp products, and have also been studied to provide pH-responsive properties to monitor chicken and shrimp freshness [10,68,92]. However, many natural pigments suffer from poor color stability and sensitivity. To improve performance, strategies such as encapsulation, formation of nanocomposites, and chemical modification of pigments or polysaccharide matrices have been employed, enhancing chromatic stability, responsiveness, and durability under real storage conditions [125].

**Figure 4:**
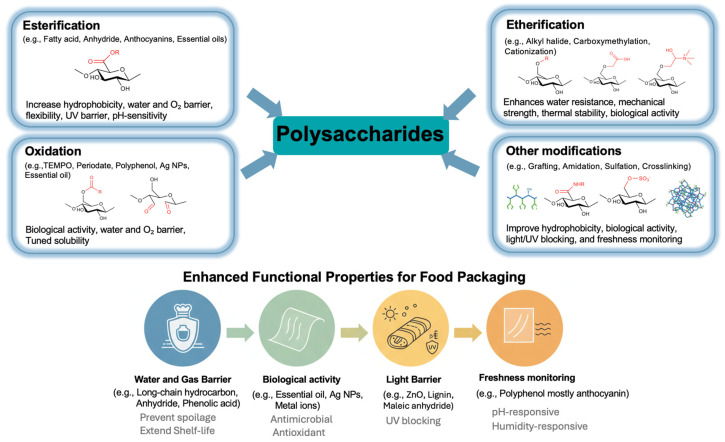
Conceptual summary showing how different modification routes improve polysaccharide properties and how combined modifications further enhance barrier performance, biological activity, and freshness-monitoring functions.

Overall, chemical modification plays a critical role in tailoring polysaccharides to meet the functional requirements of modern food packaging. Across esterification, oxidation, etherification, amidation, and other modification strategies, significant enhancements have been achieved in mechanical strength, water and gas barrier properties, hydrophobicity, thermal stability, and active functionalities such as antioxidant and antimicrobial activity, as well as emerging smart features like freshness monitoring (Figure 4). These findings highlight the importance of selecting modification methods that align with the intended packaging function while ensuring that biodegradability is preserved. Nevertheless, challenges remain in terms of solvent selection, catalyst cost, reaction efficiency, and downstream processing (e.g., purification, solvent recovery, and drying), which may limit large-scale implementation. Future research should therefore place greater emphasis on the development of cost-effective and low-toxicity reagents, efficient solvent recovery strategies, and scalable, industrially viable processing routes. As research progresses, the strategic combination of chemical modifications is expected to yield high-performance, sustainable polysaccharide-based materials capable of replacing or complementing conventional plastics.

## 5. Preparation Techniques for Functionalized Polysaccharide Packaging Films

The performance of polysaccharide-based food packaging materials depends not only on their chemical structure but also on the fabrication methods used to convert them into films, coatings, and functional layers. A range of fabrication methods has been explored to transform modified polysaccharides into uniform, stable, and high-performance packaging materials. Each method offers distinct advantages and limitations in terms of scalability, cost, energy demand, and compatibility with various polysaccharides [126]. This section provides an overview of these fabrication strategies and highlights how processing choices shape the final properties and practical potential of polysaccharide-based packaging.

### 5.1. Solution Casting

Solution casting is one of the most widely used methods to fabricate modified polysaccharide films from chitosan, alginate, starch, pectin, and cellulose [127,128,129,130]. In this technique, polysaccharides are dissolved in a suitable solvent, optionally mixed with plasticizers or functional additives, and then poured onto a flat surface to dry, forming a uniform film. This method offers a simple and low-cost setup, making it ideal for lab-scale studies and for achieving precise control over film properties. However, slow drying times and the high energy demand of large-scale drying can limit the scalability of this method [131]. In addition, the suitability of solution casting for food packaging applications strongly depends on solvent selection. While water and food-grade solvents are generally acceptable, the use of toxic or non-food-approved organic solvents necessitates extensive solvent removal and compliance with regulatory requirements, which may further restrict practical implementation. Despite these limitations, solution casting remains a highly effective approach for producing active packaging for fruits and vegetables, as well as transparent biodegradable films for food packaging applications. Nian et al. demonstrated that chitosan-based biopolymer films produced using the solution casting method with water as the solvent extended the shelf life of tomatoes by 4–5 days compared with commercially packaged tomatoes (CON), which showed visible spots by the tenth day of storage. In contrast, tomatoes packaged with chitosan quaternary ammonium salt and gelatin films (CGF) developed spots on the fifteenth day, while samples packaged with the modified MOF Cer@MHKUST-1 incorporated into chitosan quaternary ammonium salt and gelatin films (CMCGF) maintained relatively unchanged morphology even on the twentieth day of storage [108].

### 5.2. Extrusion

Extrusion is widely used to process thermoplastic-modified polysaccharides, including thermoplastic starch-based and polylactic acid-based composites [132,133,134]. In this method, polymer blends are forced through a die under controlled heat and pressure to produce continuous films or sheets with uniform dimensions. A key advantage of extrusion is its industrial scalability, supporting continuous, high-throughput production with minimal labor. It is the dominant processing method in commercial film manufacturing, particularly through blown-film and cast-film extrusion. The process allows precise control by adjusting parameters such as temperature, feed rate, and screw speed, and it accommodates compounding steps including mixing, plasticization, and incorporation of fillers or active agents within a single continuous operation [134]. Extrusion is also energy-efficient at scale and readily integrates into existing packaging production lines. However, the elevated temperatures involved may degrade heat-sensitive polysaccharides or bioactive additives, and only materials with thermoplastic behavior or adequate plasticization can be effectively processed. Equipment costs are also higher than those of small-scale methods, such as solution casting [131]. Despite these challenges, extrusion has been successfully applied to produce biodegradable active films for sustainable food packaging. For example, an extrusion-blown oxidized starch/poly(butylene adipate-co-terephthalate) (OST–PBAT) composite film extended the shelf life of chilled pork by two days compared with conventional polyethylene (PE) films [132].

### 5.3. Electrospinning and Electrospraying

Electrospinning and electrospraying are electrohydrodynamic techniques used to fabricate ultrafine fibers and micro/nanoparticles from polysaccharide solutions under a high-voltage electric field. Common electrospun polysaccharides include chitosan, cellulose, starch, and alginate derivatives [135]. In electrospinning, the applied voltage overcomes surface tension, drawing a continuous polymer jet that solidifies into nanofibers, while electrospraying produces droplets that form micro- to nanoscale particles. These methods enable the creation of highly porous, lightweight structures with large surface area, and the incorporation of antioxidants and antimicrobials makes it particularly promising for active and intelligent packaging [136]. However, electrospinning requires careful optimization of solution conductivity, viscosity, and voltage parameters, and many polysaccharides need chemical modification or blending to achieve stable fiber formation. Despite these constraints, electrospinning and electrospraying are increasingly explored for antimicrobial membranes, antioxidant-release systems, and biodegradable nanofiber coatings, demonstrating strong potential for next-generation functional food packaging [137]. For example, a cellulose-based material was coated using electrospraying onto paper, which was then used to preserve pears. After 90 days of storage, pears stored in the cellulose active packaging retained better color and texture, ripened later, exhibited lower microbial loads, and received higher scores in sensory evaluation compared with pears wrapped in uncoated paper or left unpackaged [138].

### 5.4. Coating

Coating is a versatile method in which polysaccharide solutions or dispersions are applied directly onto food surfaces or packaging substrates to form thin functional layers. Coating methods, such as dip-coating and spray-coating, are commonly used for applying thin functional layers of chitosan, alginate, cellulose and cellulose derivatives [139]. These polysaccharides readily form smooth aqueous dispersions that dry into compact films, restricting the transfer of O_2_, H_2_O, and CO_2_, thereby helping to slow microbial spoilage. Coating methods allow for the efficient incorporation of bioactive compounds and are widely used to produce edible coatings. Their simplicity, low cost, and compatibility with existing industrial equipment make them appealing. However, achieving strong adhesion and uniform layer thickness remains challenging. Despite these limitations, coatings are extensively applied to fruits, vegetables, and paper-based packaging to enhance barrier, antimicrobial, and antioxidant performance. For instance, an alginate-based edible film extended the shelf life of tomatoes up to sixteen days without visible defects, owing to the excellent physical strength, UV-blocking capability, and antimicrobial activity of the optimized alginate/AV/ZnO-NPs formulation [140].

In summary, a variety of fabrication methods have been developed to convert chemically modified polysaccharides into functional films and coatings for food packaging. Solution casting and coating techniques offer simplicity and flexibility for lab-scale or specialized applications, while extrusion enables scalable production of continuous films and multilayer structures. Spray-drying and electrospinning provide additional versatility, allowing encapsulation of bioactive compounds and the creation of nanofibrous mats with high surface area for active packaging. 3D printing is also emerging as a promising fabrication strategy for polysaccharide-based packaging, particularly for customized designs and functional structures [141]. By using extrusion-based printing of polysaccharide gels or modified biopolymer inks, complex geometries and multilayer architectures can be produced with precise control over thickness, porosity, and spatial distribution of active compounds [142]. This approach may enable on-demand fabrication of packaging components tailored to specific food products or freshness-monitoring functions. However, current limitations include the poor processability and limited solubility of biopolymers in water or common organic solvents [141]. Therefore, the development of new solvent systems is necessary to support biopolymer processing and 3D printing. Each method presents distinct advantages and limitations, and the choice of fabrication technique depends on the desired film properties, end-use application, and scalability requirements. By carefully selecting and optimizing these processing strategies, polysaccharide-based materials can be effectively translated from laboratory research to sustainable, multifunctional food packaging solutions. Table 5 summarizes the relationships between polysaccharide type, representative chemical modification routes, fabrication strategies, and typical food packaging applications.

## 6. Challenges and Future Perspectives

Despite significant progress in the chemical modification of polysaccharides for food packaging, several scientific, environmental, economic, and regulatory barriers still restrict their large-scale implementation. Many modified polysaccharides exhibit enhanced mechanical strength, barrier performance, and functional properties; however, concerns regarding food safety, environmental sustainability, production cost, and overall economic feasibility continue to pose challenges [157]. Additionally, performance limitations under real-world storage and processing conditions remain a major hurdle before these materials can reliably replace conventional plastics. Looking forward, advances in green chemistry, scalable manufacturing, and multifunctional design will be crucial to unlocking the full potential of chemically modified polysaccharides in sustainable packaging systems.

### 6.1. Food Safety

Food safety is a critical consideration when introducing polysaccharides into food-packaging applications. While unmodified polysaccharides that are already part of the food chain, such as starch, generally do not require extensive safety testing due to their well-established consumption history [158], modified materials must undergo rigorous evaluation, which can add several years to the development timeline. Chemical modifications can create new structures or reactive groups, or leave residual reagents, unreacted monomers, or byproducts that could potentially migrate into food and affect its safety and quality. Minimizing such risks requires cleaner reaction systems, greener reagents, improved purification processes, and the development of materials with inherently reduce migration potential. In addition to general toxicological evaluation, food-contact approval requires demonstrating compliance with migration limits under intended-use conditions. This includes overall migration and specific migration testing, particularly for residual reagents, unreacted monomers, catalysts, and low-molecular-weight byproducts that may form during modification [159]. Current safety assessments often rely on in vitro assays, which provide useful preliminary data but may not fully represent complex biological responses. More advanced evaluation models, such as in vivo testing and emerging platforms like quantitative adverse outcome pathway, can offer more realistic insights into the long-term safety of modified polysaccharides [160]. Strengthening these evaluation frameworks and aligning them with international regulatory standards, such as the US Food and Drug Administration (FDA) Food Contact Materials regulations and the European Union’s Framework Regulation (EC) No. 1935/2004 (Food Contact Regulation), will be essential for ensuring that these materials meet safety requirements before entering the food packaging market [161]. Moreover, risk assessment should consider NIASs (non-intentionally added substances), which may arise from side reactions, impurities, or degradation during processing and storage. Therefore, safety testing should be supported by appropriate analytical methods and performed using food simulants under relevant time–temperature conditions.

### 6.2. Environmental and Sustainability Considerations

Environmental sustainability is a key motivation for developing polysaccharide-based packaging, yet chemical modification can introduce challenges that must be carefully addressed. Life cycle assessment (LCA) provides a systematic framework to evaluate the environmental impact of polysaccharide-based packaging, considering factors such as raw material sourcing, energy consumption, solvent use, chemical reagents, and end-of-life disposal [162]. While native polysaccharides are biodegradable and derived from renewable resources, certain modification processes may rely on harsh chemicals, organic solvents, or energy-intensive conditions that reduce overall environmental benefits. Adopting green reaction systems, minimizing solvent use, and prioritizing reagents that do not compromise environmental performance are essential strategies [41]. Integrating renewable feedstocks, improving energy efficiency, and designing modifications that maintain or enhance biodegradability are critical for aligning polysaccharide-based packaging with sustainable packaging goals.

### 6.3. Biodegradability

Biodegradability is a defining feature of polysaccharide-based packaging, ensuring that materials break down naturally without leaving persistent pollutants. While chemical modifications can enhance functional performance, they may also influence degradation rates and pathways. Studies on functionalized cellulose and other polysaccharides have shown that it is possible to retain mechanical strength, barrier properties, and other functional benefits while still allowing microbial decomposition under composting or soil conditions [163].

A preliminary study demonstrated that cellulose functionalized with palmitic acid did not degrade significantly over twelve weeks under composting conditions [164], unlike cellulose acetate. More recently, however, Entwistle et al. demonstrated that this functionalized cellulose polymer could be broken down by the judicious use of both a lipase (to cleave the functional group) before a cellulase can attack the carbohydrate linkages, solubilizing the polymer [165]. The authors also demonstrated extensive polymer degradation in a mimicked bird’s gut, simulated using pepsin and pancreatic enzymes at controlled pH to mimic real ingestion conditions, and finally, by the whole-cell soil fungus *Mucor* sp., commonly found in soil and compost environments. These findings highlight that, through the thoughtful selection of modification strategies, functional packaging materials can achieve a balance between enhanced performance and biodegradability, supporting circular economy principles and sustainable food packaging solutions.

From a circular design perspective, future modification strategies should be selected not only to enhance in-use performance but also to enable predictable end-of-life degradation. This includes designing cleavable linkages, avoiding highly persistent hydrophobic substituents, and prioritizing functional groups that can be removed or degraded under mild composting or soil conditions. Such “design-for-deconstruction” approaches can help ensure that improved functionality does not compromise compostability or circularity.

### 6.4. Production Cost and Economic Feasibility

High production costs remain a significant barrier to the commercialization of chemically modified polysaccharides for food packaging. Many modification processes require purified feedstocks, specialty reagents, controlled reaction conditions, and extensive purification steps, all of which increase overall manufacturing expenses. Compared with conventional petroleum-based plastics, these materials are often economically uncompetitive. Strategies to reduce costs include developing more efficient and scalable reaction methods, using renewable or waste biomass as raw materials, minimizing solvent and energy consumption, and employing process simulation and optimization tools, such as Aspen Plus, to optimize chemical usage [41]. Addressing these economic challenges is essential to make polysaccharide-based packaging viable for large-scale, sustainable applications.

### 6.5. Performance Limitations in Real-World Conditions and Future Directions

Although chemically modified polysaccharides show promising properties in the laboratory, their performance under real-world conditions can be limited. High moisture sensitivity reduced mechanical strength under humid conditions, and insufficient barrier performance against gases or oils can restrict practical applications. Thermal instability may also limit compatibility with common food-processing or packaging operations. Addressing these challenges requires the development of multifunctional modifications, blending with other biopolymers, or forming composites to maintain stability and performance in diverse storage and processing environments. Future research should focus on combining green chemistry approaches with scalable manufacturing methods to produce cost-effective, multifunctional polysaccharide-based packaging. Integrating bioactive compounds, nanomaterials, or hybrid structures can further enhance barrier, mechanical, antioxidant, and antimicrobial properties. A key goal is to develop materials that can degrade easily after use and under mild conditions, without requiring industrial composting facilities or complex treatment processes [166]. In addition, many studies still lack microbiological validation and shelf-life assessment of polysaccharide-based food packaging films under realistic storage conditions. These studies should include evaluating antimicrobial performance, monitoring microbial growth on packaged foods, and conducting storage tests to confirm shelf-life extension. Safety assessment is also essential, including migration testing and evaluation of potential risks associated with residual reagents or incorporated active compounds [167]. Standardized safety assessments, life cycle analysis, and regulatory alignment will be critical for commercialization. Advances in these areas will enable polysaccharide-based materials to compete with conventional plastics while meeting sustainability and functional requirements for modern food packaging.

## 7. Conclusions

Chemically modified polysaccharides represent a promising and sustainable alternative to conventional petroleum-based plastics in food packaging. Advances in chemical modification techniques, including esterification, oxidation, amidation, etherification, and grafting, have enabled the development of materials with improved mechanical strength, enhanced barrier properties against water, oxygen, and fats, and additional functionalities such as antioxidant, antimicrobial, and hydrophobic characteristics. These modifications expand the applicability of polysaccharides and offer potential for active and intelligent packaging solutions that can prolong shelf life and maintain food quality. Despite these advances, challenges such as food safety, environmental impact, production cost, scalability, and performance under real-world conditions remain. Future research should focus on greener modification methods, such as enzymatic treatments, solvent-free or aqueous-based reactions, and the use of non-toxic or renewable reagents, alongside cost-effective and scalable production, to develop materials that are readily degradable under mild or ambient conditions. By integrating innovative chemical strategies, sustainable manufacturing approaches, and multifunctional design, chemically modified polysaccharides have the potential to meet the demanding requirements of modern food packaging while supporting eco-friendly, safe, and intelligent packaging solutions, ultimately contributing to global sustainability and reducing plastic pollution.

## Figures and Tables

**Table 3:** Representative studies on etherified polysaccharides: compounds incorporated, modification method, functional improvements, advantages, and challenges.

Polysaccharides	Secondary Compound	Modification Method	Functional Properties	Advantages	Challenges	Reference
Cellulose nanocrystals (CNCs)	EPTMAC *, Carboxymethyl cellulose	CNCs cationized through alkali-activated etherification with EPTMAC	Improved thermal stability and tensile strength	Sustainability, odorless, and non-toxic	Processability related to viscosity	[97]
Micro-fibrillated cellulose (MFC)	Propargyl bromide, allyl bromide and propyl bromide	Alkylation of MFC with alkyl bromides (NaOH-activated)	Improve water resistance and mechanical properties	Eco-friendly and cost-effective process	Dispersion and homogeneity	[91]
Carboxymethyl cellulose (CMC)	Polyvinyl alcohol (PVA), rose anthocyanin extracts	Casting of CMC/PVA film with rose anthocyanin extract	pH-responsiveness	Food-grade pH indicators	High extract concentrations, high water vapor permeability	[98]
Pectin/carboxymethyl cellulose (CMC)	Anthocyanins, metal ions (Zn^2+^, Mg^2+^, and Ca^2+^)	Metal-ion crosslinking of pectin/CMC and complexation with anthocyanins	Antioxidant capacity	Freshness monitoring	Limited visual detection	[92]
Methyl cellulose (MC)	Walnut green husk polyphenols (WGHP), curdlan (CD)	WGHP was added to a CD–MC matrix via solution casting	Antimicrobial and antioxidant	Prolonged the shelf-life of fried walnuts	Instability	[93]
Carboxymethyl cellulose (CMC)	Gelatin, pomegranate peel extract (PPE)	PPE was added to a CMC–gelatin film matrix via solution casting	Antimicrobial and antioxidant	Prolonged the shelf-life of beef	Enhanced surface hydrophilicity when adding extract	[94]
Carboxymethyl chitosan (CMCS)	Hydroxyethyl cellulose (HEC), ZnO nanoparticles	HEC/CMCS film incorporated with ZnO nanoparticles via solution casting	Antibacterial activity and UV-blocking	Improved water resistant	Reduction in tensile strength (from 4.18 to 2.1 MPa)	[95]
Carboxymethyl chitosan (CMCS)	Vanillin, ZnO	Schiff-base reaction of vanillin with CMCS and Zn^2+^ complexation	Antibacterial activity	Prolonged the shelf-life of mango, biodegradable	Reduction in strain (from 304% to 163%)	[96]
Carboxymethyl starch (CMS)	κ-carrageenan, gum ghatti	κ-carrageenan/CMS/gum ghatti composite film prepared via solution casting	Reduction in water solubility, decrease in water vapor permeability	Edible coffee packaging	Short-term moisture protection under high humidity (~6 h)	[99]
hydroxypropyl starch (HPS)	Curdlan (CD)	HPS/CD composite film via solution casting	Reduction in water solubility	Excellent mechanical strength	Further studies needed for real food systems.	[100]

* EPTMAC = 2,3-epoxypropyl trimethylammonium chloride.

**Table 4:** Examples of alternative modifications of polysaccharides: compounds incorporated, modification method, potential advantages, and challenges in food packaging applications.

Polysaccharides	Other Compounds	Modification Method	Functional Properties	Advantages	Challenges	Reference
Alginate	Alkyl amines (C_8–18_)	Amidation of alginate with alkyl amines, activated by CMPI or EDC	Improve hydrophobicity	Bio-based materials	Limited processability (no melting point)	[50]
Alginate	Amino acids	Amidation of alginate with Amino acids, activated by CMPI	Improve hydrophobicity	Bio-based materials	Further studies needed for degradability	[101]
Chitosan	Vanillic acid (VA)	Amidation and esterification of chitosan and VA, activated by EDC/NHS	Improve water vapor barrier, antioxidant, and antimicrobial properties	Cherry tomato preservation	Increased swelling degree and water solubility	[102]
Cellulose acetate (CA)	Alginate/carrageenan	Blending alginate or carrageenan into CA via solvent casting	Improve the tensile strength, thermal stability, and antibacterial activity	Biodegradable	Decrease in elongation-at-break	[103]
Chitosan	κ-carrageenan	Blending chitosan/κ-carrageenan via solvent casting	Improve hydrophobicity, higher flexibility, smooth and uniform surface	Bio-based materials	Thermal degradation during casting	[104]
Alginate	Lignin	Blending lignin and alginate via solvent casting	Improved opacity and physical properties	Light barrier and antimicrobial properties	Reduction in tensile strength	[105]
Microcrystalline cellulose (MCC)	Rosin	Rosin-modified MCC films via hydroxylyne click grafting chemistry	Improve water and oxygen barrier, UV-blocking	Biodegradability	Production cost from ionic liquid use	[106]
Chitosan	Starch, glutaraldehyde	Quaternization of Chitosan, blended with starch, and crosslinked using glutaraldehyde (Schiff-base reaction)	Antimicrobial properties	Meat preservation	For quaternary ammonium modification, unreacted reagent components may remain in the product, posing risks of toxicity or allergic reactions.	[61]
Chitosan	Polyvinyl alcohol (PVA)	Quaternization of chitosan and blended with PVA, using solution casting	Antimicrobial properties	Strawberry preservation		[107]
Chitosan	Gelatin and metal–organic skeletons (MOF- Cer@MHKUST-1)	Quaternization of chitosan and blended with gelation and MOF	Improve water vapor barrier, antimicrobial properties	Tomato preservation		[108]
Chitosan	Cinnamaldehyde (Cin)	Cin was incorporated through chemical grafting (imine formation with chitosan)	Humidity/pH dual-responsive, antimicrobial properties	Strawberry preservation	-	[109]
κ-carrageenan	Agar, ZnO, TiO_2_, Clitoria ternatea Linn anthocyanin	Sol–gel casting of bilayer films	pH indication, light barrier	Pork freshness monitoring	Color stability decreases as temperature increases	[110]

CMPI = 2-chloro-1-methylpyridinium iodide. EDC = 1-ethyl-3-(3-dimethylaminopropyl) carbodiimide. NHS = N-Hydroxysuccinimide.

**Table 5:** Summary of relationships between polysaccharide type, chemical modification strategy, fabrication method, and food packaging application.

Polysaccharide	Modification Method	Fabrication Method	Packaging Application/Function	References
Chitosan	Quaternization, blending with active fillers	Solution casting, coating, electrospinning/electrospraying	Antimicrobial films, active packaging for fruits/vegetables	[108,128,143,144,145]
Alginate	Amidation, composite formation	Solution casting, coating, electrospinning	Edible coatings, antimicrobial and UV-blocking films	[101,135,140]
Starch	Esterification, crosslinking, blending	Extrusion, solution casting, coating	Antimicrobial films, biodegradable films, meat packaging	[127,128,132,146]
Cellulose/cellulose derivatives	Etherification, oxidation, blending	Solution casting, electrospinning/electrospraying, coating	Edible films, pear packaging, antimicrobial active coating	[138,139,147]
Pectin	Blending, crosslinking	Solution casting, extrusion, coating	Edible film and coating, fruit/vegetable shelf-life extension, packaging film	[148,149,150,151]
Carrageenan	Blending	Solution casting, electrospinning, coating	pH-indicative films, antioxidant and antimicrobial properties, meat preservation	[152,153,154]
Agar	Esterification, blending	Solution casting, coating	UV barrier, resistant and elastic film, fish preservation	[79,155,156]

## Data Availability

No new data were created or analyzed in this study. Data sharing is not applicable to this article.
